# Genetic variants associated with sepsis-associated acute kidney injury

**DOI:** 10.1371/journal.pone.0311318

**Published:** 2024-12-05

**Authors:** Nicholas J. Douville, Lisa Bastarache, Emily Bertucci-Richter, Snehal Patil, Elizabeth S. Jewell, Robert E. Freundlich, Miklos D. Kertai, Milo C. Engoren

**Affiliations:** 1 Department of Anesthesiology, Michigan Medicine, Ann Arbor, Michigan, United States of America; 2 Institute of Healthcare Policy & Innovation, University of Michigan, Ann Arbor, Michigan, United States of America; 3 Department of Computational Medicine and Bioinformatics, University of Michigan, Ann Arbor, Michigan, United States of America; 4 Department of Biomedical Informatics, Vanderbilt University Medical Center, Nashville, Tennessee, United States of America; 5 Precision Health, University of Michigan, Ann Arbor, Michigan, United States of America; 6 Department of Anesthesiology, Vanderbilt University Medical Center, Nashville, Tennessee, United States of America; Cairo University Kasr Alainy Faculty of Medicine, EGYPT

## Abstract

**Background:**

Kidney dysfunction is a common complication in septic patients. Studies have identified numerous risk factors for sepsis-associated acute kidney injury (S-AKI), yet there is wide variability in the incidence even among patients with similar risk factors, suggesting the presence of additional uncharacterized risk factors, including genetic differences. The expansion of biobanks, advances in genotyping, and standardized diagnostic criteria have enabled large-scale, hypothesis-generating studies into the genetic mechanisms underlying S-AKI. We hypothesize that the genetic pathway behind S-AKI has overlapping mechanisms with key differences based upon the specific subtype of acute kidney injury (AKI).

**Methods:**

To test this hypothesis, we performed a genome-wide association study (GWAS) of S-AKI in three logistic regression models. Model 1, controlled for 1) age, 2) sex, 3) genotyping chip, and 4) the first five principal components. In Model 2, pre-sepsis baseline serum creatinine was added to the variables in Model 1. Finally, in Model 3, we controlled for the full range of patient, clinical, and ICU-related risk factors. Each of the 3-models were repeated in a pre-specified sensitivity analysis of higher severity S-AKI, defined as KDIGO Stage 2 or 3. We then compare associated variants and genes from our GWAS with previously published AKI sub-types and model other factors associated with S-AKI in our dataset.

**Findings:**

3,348 qualifying Sepsis-3 patients have been genotyped in our dataset. Of these patients, 383 (11.4%) developed Stage 1, 2, or 3 AKI (primary outcome) and 181 (5.4%) developed Stage 2 or 3 AKI (sensitivity analysis). The median age was 61 years (interquartile range (IQR): 51,69), 42% were female, and the increase in SOFA score (between 48-hours before to 24-hours after the onset of suspected infection) was 2 (2–3). No variants exceeded our threshold for genome-wide significance (*P*<5x10^-8^), however, a total of 13 variants exceeded the suggestive (*P*<1x10^-6^) threshold. Notably, **rs184516290** (chr1:199814965:G:A), near the ***NR5A2*** gene, chr1:199805801:T:TA, also near the ***NR5A2*** gene, and **rs117313146** (chr15:31999784:G:C), near the ***CHRNA7*** gene, were associated with S-AKI at the suggestive level in all three models presented. Variants in the suppressor of fused homolog (***SUFU***) gene, previously shown to be correlated with renal function in bacteremic patients, consistently exceeded the *P*<0.05 threshold in our models.

**Conclusions:**

While failing to identify any novel association for S-AKI at the level of genome-wide significance, our study did suggest multiple variants in previously characterized pathways for S-AKI including ***CHRNA7*, *NR5A2*,** and ***SUFU***. We failed to replicate associations from multiple prior studies which may result from differences in how the phenotype was defined or, alternatively, limited genetic contribution and low heritability.

## Background

Sepsis is a life-threatening organ dysfunction resulting from dysregulated response to infection, [1] and despite decades of research remains a leading causes of morbidity and death in hospitalized patients [2]. Kidney dysfunction occurs in 30–50% of septic patients [3–5] leading to sepsis-associated AKI (S-AKI) [6]. S-AKI is associated with increased hospital stay, greater resource consumption, and increased mortality, [7–10] even in less severe cases of S-AKI not requiring dialysis [5].

Studies have identified numerous risk factors for S-AKI, including male sex, older age, comorbidities including hypertension and diabetes, source and type of infection, and angiotensin converting enzyme inhibitors or angiotensin receptor antagonists use [11–13]. More severe sepsis, as marked by shock, receipt of vasopressors, or mechanical ventilation, is also a risk factor for S-AKI [12, 13]. Yet there is wide variability in the incidence of AKI, even among patients with similar risk factors, suggesting the presence of additional uncharacterized risk factors, such as genetic differences [14]. The genetics of AKI had been traditionally studied in a hypothesis-driven manner, testing a limited number of candidate polymorphisms [15]. The expansion of biobanks and recent advances in genotyping have enabled large-scale, hypothesis-generating studies into the genetic mechanisms underlying AKI phenotypes, including surgical [16, 17] and intensive care populations [18]. Furthermore, standardized definitions for both sepsis (*Sepsis-3*) [1] and AKI (*Kidney Disease*: *Improving Global Outcomes*) [19] improve the breadth and generalizability of phenotypes that can be curated and studied. A recent study, characterizing genetic differences resulting from transition between the Sepsis-2 to Sepsis-3 diagnostic criteria, showed 16% of variants remained whether either definition was employed, while 49% and 35% were isolated in the Sepis-2 and Sepsis-3 cohorts, respectively [20]. Large-scale, unbiased investigations on S-AKI using standardized definitions for sepsis (Sepsis-3) and AKI (KDIGO) are lacking [15].

We hypothesize that the genetic pathway behind S-AKI has overlapping mechanisms with key differences based upon the specific subtype of AKI. To test this hypothesis, we performed a genome-wide association study (GWAS) of S-AKI in a large, single-center biorepository of septic patients [13, 20] with linked genotype and electronic health records [16, 17]. We then compare associated variants and genes from our GWAS with previously published AKI sub-types and model other factors associated with S-AKI in our dataset.

## Methods

### Study design and setting

**Fig 1:**
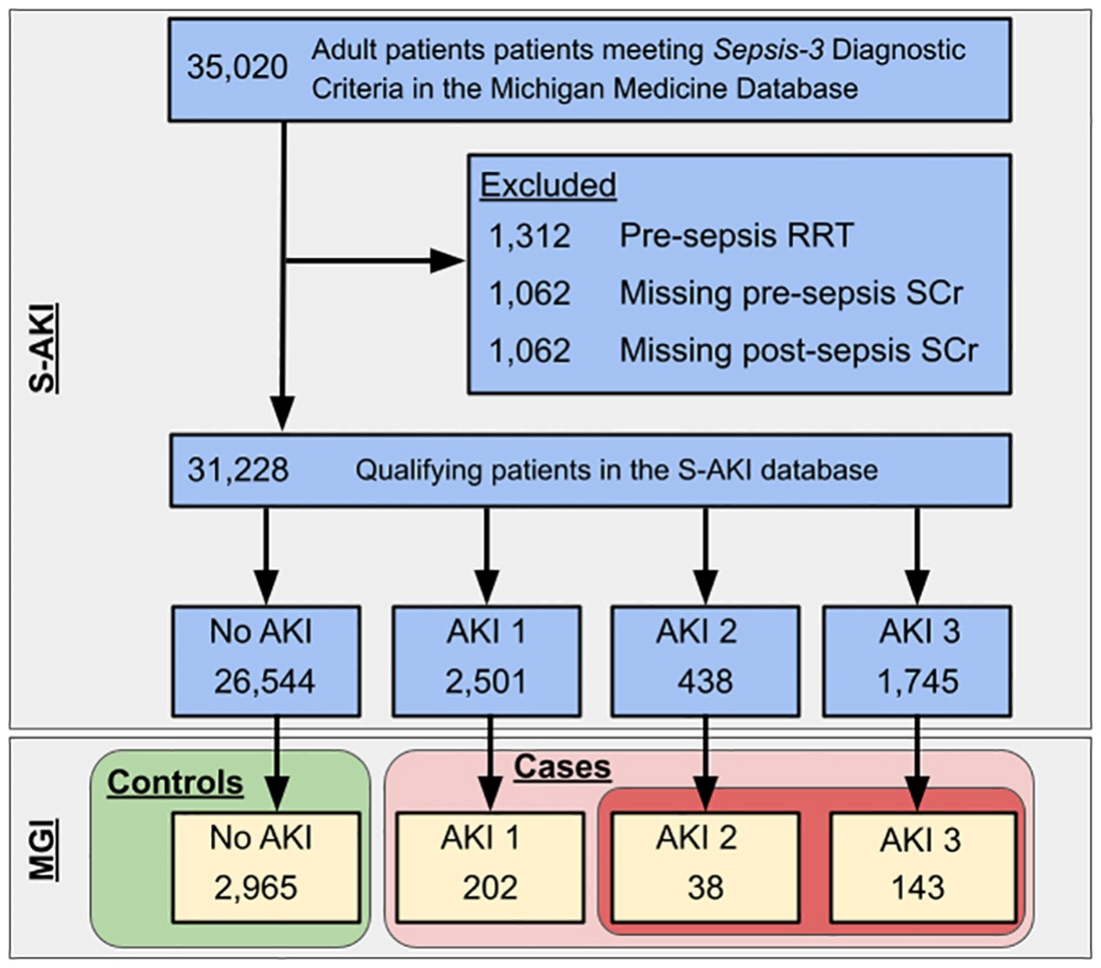
Derivation of study cohort. Abbreviations: AKI = Acute Kidney Injury; AKI 1 = Kidney Disease: Improving Global Outcome (KDIGO) Stage 1 AKI; AKI 2 = KDIGO Stage 2 AKI; AKI 3 = KDIGO Stage 3 AKI; MGI = Michigan Genomics Initiative; RRT = Renal Replacement Therapy; S-AKI = Sepsis-associated Acute Kidney Injury; SCr = Serum Creatinine; Sepsis-3 = The Third International Consensus Definitions for Sepsis and Septic Shock.

This study was approved by the Institutional Review Board (HUM00126162). We used the STREGA checklist when writing our report [21]. All participants provided written informed consent. Our study cohort was created from the intersection between two previously described sources: (i) Michigan Medicine S-AKI dataset [13, 22] (N = 35,020 patients) and (ii) MGI biobank (*Data Freeze 6*, N = 80,529 patients). General inclusion criteria were adult patients treated at Michigan Medicine between July 10, 2009 and September 7, 2019 with genotype available. Data were accessed for research purposes September 12, 2023. Patients were excluded if they required renal replacement therapy prior to sepsis, were missing baseline creatinine, or had no follow-up creatinine (Fig 1).

### Outcome measure and data collection

The outcome of interest was Stage 1, 2, or 3 AKI, defined per KDIGO guidelines, [19] within 7-days of sepsis onset, defined by Sepsis-3 criteria [1]. Baseline creatinine was defined as the most recent value at or before sepsis onset. The control population was patients with no AKI, within 7-days of developing sepsis. Sepsis-3 criteria were determined from the S-AKI dataset, which included vital signs, laboratory results, microbiology results, antibiotic use, and receipt of dialysis, based upon a previously described methodology [13]. A pre-specified sensitivity analysis was performed on higher severity (KDIGO, Stage 2 or 3) cases of S-AKI. In this sensitivity analysis, Stage 1 AKI was omitted from the analysis (ie: considered neither “case,” nor “control’).

### Selection criteria for sepsis database

The sepsis database was created by querying (DataDirect, University of Michigan, Ann Arbor, MI) the data warehouse for all patients meeting Sepsis-3 criteria between July 10, 2009 and September 7, 2019, as previously described [13]. In brief, the data warehouse contains demographics, vital signs, laboratory results, microbiology results, antibiotic use, operation, and processes of care, including receipt of vasopressors, mechanical ventilation, kidney replacement therapy, and blood transfusion. Patients who (i) received acute kidney replacement therapy in the 14 days prior to sepsis, (ii) required chronic dialysis, (iii) were missing baseline creatinine, or (iv) had no follow-up creatinine were excluded. Patients met Sepsis-3 criteria if they had suspected infection as documented by blood cultures, received antibiotics within the time window from 24 hours before to 72 hours after blood cultures were obtained, and had ≥ 2 points increase in Sequential Organ Failure Assessment (SOFA) score between 48-hours prior to 24-hours after onset of suspected infection. The timing of suspected infection was defined by antibiotic administration or obtaining cultures (whichever occurred first). The cardiovascular component of the SOFA score was calculated including vasopressin (0.4 U/min vasopressin = 0.1 mcg/kg/min norepinephrine) [23]. Patients who had Kidney Disease Improving Global Outcomes (KDIGO) Stage 1, 2 or 3 acute kidney injury (AKI) were compared to patients without any AKI.

As urine output was not reliably obtained in all patients, only the creatinine criteria were used. Stage 2 AKI was defined as serum creatinine 2–2.9 times baseline value; Stage 3 as serum creatinine ≥ 3 times baseline value, a rise to ≥ 4.0 mg/dL, or initiation of kidney replacement therapy. No AKI was defined as any rise in serum creatinine <0.3 mg/dL within 48 hours and absence of ≥ 50% within 7 days.

### Genotype

The Michigan Genomics Initiative (MGI) is a biobank that collects blood or saliva samples from adult patients receiving care at Michigan Medicine. In brief, MGI patients were genotyped on customized Illumina Human-CoreExome (v1.0, v1.1, v1.3) or Illumina Infinium Global Screening Arrays (v1.3) (Illumina, Inc. San Diego, CA) and subsequently imputed to the Trans-Omics for Precision Medicine (TOPMed) reference panel using the TOPMed Imputation Server [24]. Variants with R^2^ ≥ 0.3 and minor allele frequency (MAF) ≥ 0.01% were selected, resulting in more than 52-million imputed variants after quality control and filtering. The European population was defined using the majority ADMIXTURE global ancestry fraction (Q-value). There were not enough patients of non-European ancestry (enrolled in Michigan Genomics Initiative) to perform a more comprehensive analysis in cohorts of non-European ancestry, therefore, our study population was restricted to patients of European descent. Additional genotype characterization and quality control from MGI is described in S1 Appendix.

### Covariates

Demographic, comorbidity, laboratory, and exposure data were derived from the S-AKI database. Specific pre-sepsis comorbidities were obtained from *International Classification of Diseases*-9 and -10 based upon grouping schema from the Elixhauser comorbidity index [25].

### Data analysis

Perioperative and intraoperative characteristics were summarized using medians and interquartile ranges for continuous variables and counts and percentages for categorical covariates. Comparisons of continuous data were made using Mann-Whitney *U* Tests and categorical data were compared using Pearson chi-square tests. *P*-value < 0.05 was selected *a priori* to denote statistical significance.

GWAS was performed using the *Scalable and Accurate Implementation of Generalized mixed model* (SAIGE) logistic regression model with kinship adjustment on genetic data from a European cohort in MGI Freeze 6 [26]. The European population was defined using the majority ADMIXTURE global ancestry fraction (Q-value). For association tests we used TOPMed Imputed genotypes and excluded variants with low imputation quality (Rsq < 0.8) or very rare minor alleles (minor allele frequency < 0.1%). Genome-wide significance threshold (*P* < 5 × 10^−8^) was selected *a priori* for identification of novel loci and a lower threshold was selected as a *suggestive* threshold (and for *replication* of previously reported loci) (*P* < 1 × 10^−6^) [27]. Genome-wide association was assessed in three logistic regression models, as previously described [16, 17]. Model 1, controlled for 1) age, 2) sex, 3) genotyping chip, and 4) the first five principal components (PCs). In Model 2, pre-sepsis baseline serum creatinine was added to the variables in Model 1. Finally, in Model 3, we controlled for the full range of patient, clinical, and ICU-related risk factors. Each of the 3-models were repeated in a pre-specified sensitivity analysis of higher severity S-AKI, defined as KDIGO Stage 2 or 3.

Results of the GWAS were visualized and top hits annotated using the *gwasTools* repository (https://github.com/ilarsf/gwasTools) with the option to set a suggestive significance threshold (*—sigthreshold*) of 1x10^-6^. The Manhattan plot was annotated with quasi-independent top hits by creating 1Mb regions centered around variants with p-values less than 1x10^-6^ and combining overlapping intervals.

### Differential tissue enrichment

We used FUMA to test for enrichment of differential expression across tissues for the eight candidate genes suggested by our study [28]. We compared expression levels of these with 57,241 background genes. Our analysis used the GENE2FUNC function with GTEx version 8 and tested 30 general tissue types. *P*-values for the enrichment were corrected using a false discovery rate.

## Results

3,348 qualifying Sepsis-3 patients from the S-AKI database have been genotyped in MGI (Fig 1). Of these patients, 383 (11.4%) developed AKI (primary outcome) and 181 (5.4%) developed Stage 2 or 3 AKI. The median age was 61 years (IQR: 51,69), 42% were female, and median SOFA score at time of Sepsis-3 diagnosis was 2 (2,4). Additional details on the cohort can be found in Table 1.

**Table 1:** Characteristics of full cohort and univariate analyses of patients developing AKI (cases) versus those without AKI (controls).

		Full cohort (n = 3,348)	No AKI (n = 2,965)	AKI (n = 383)	*P*-value
		N (%); Median (IQR)			
**Model 1**					
Age, years		61 (51,69)	61 (51,69)	62 (51,71)	0.0997
Female Sex		1,406 (42.0)	1,276 (43.0)	130 (33.9)	0.0008
**Model 2**					
Baseline Serum Creatinine (mg/dL)		1.0 (0.7, 1.5)	1.0 (0.7, 1.4)	1.8 (1.0, 4.0)	<0.0001
**Model 3**					
Lactate (at time of sepsis) (mmol/L)		1.0 (1.0, 1.5)	1.0 (1.0, 1.5)	1.0 (1.0, 1.7)	0.0739
Total SOFA points with Vasopressin		2 (2, 4)	2 (2, 3)	3 (2, 5)	<0.0001
Elixhauser Comorbidity Index	Complicated Diabetes	565 (16.9)	465 (15.7)	100 (26.1)	<0.0001
	Uncomplicated Diabetes	1,168 (34.9)	1,015 (34.2)	153 (39.9)	0.0314
	Congestive Heart Failure	882 (26.3)	735 (24.8)	147 (38.4)	<0.0001
	Liver Disease	1,082 (32.3)	960 (32.4)	122 (31.9)	0.8821
	Peripheral Vascular Disease	1,163 (34.7)	1,008 (34.0)	155 (40.5)	0.0144
	COPD	1,310 (39.1)	1,150 (38.8)	160 (41.8)	0.2835
	Cardiac Arrhythmias	2,152 (64.3)	1,868 (63.0)	284 (74.2)	<0.0001

AKI = Acute kidney injury; COPD = chronic obstructive pulmonary disease; dL = deciliters; L = liters; kg = kilograms; mg = milligrams; mmol = millimole; SOFA = Sequential Organ Failure Assessment

On univariate analysis, patients developing S-AKI tended to be male (66.1% in the AKI group compared to 57.0 in the No AKI group, *P* = 0.0008) (Table 1). Additionally, patients with comorbidities, including diabetes, peripheral vascular disease, and cardiac arrhythmias had a greater risk of developing S-AKI.

### Genome-wide association studies

#### Primary outcome

**Fig 2:**
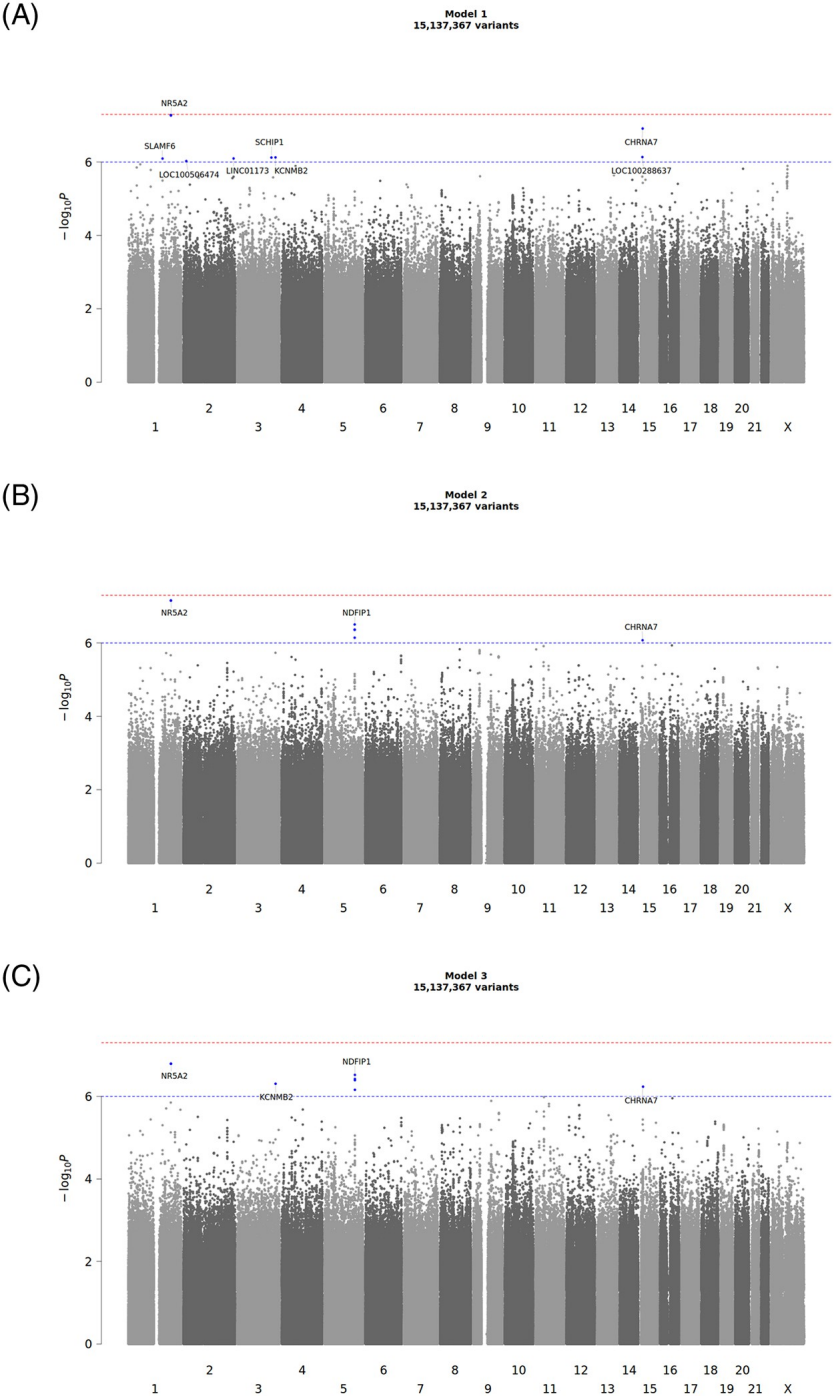
Manhattan plots for GWAS model. 2A. Model 1. 2B. Model 2. 2C. Model 3. (•) Genome Wide Significance Threshold (*P*<5x10^-8^) red dashed line. (•) Suggestive/Replicative Significance Threshold (*P*<1x10^-6^) blue dashed line.

**Fig 3:**
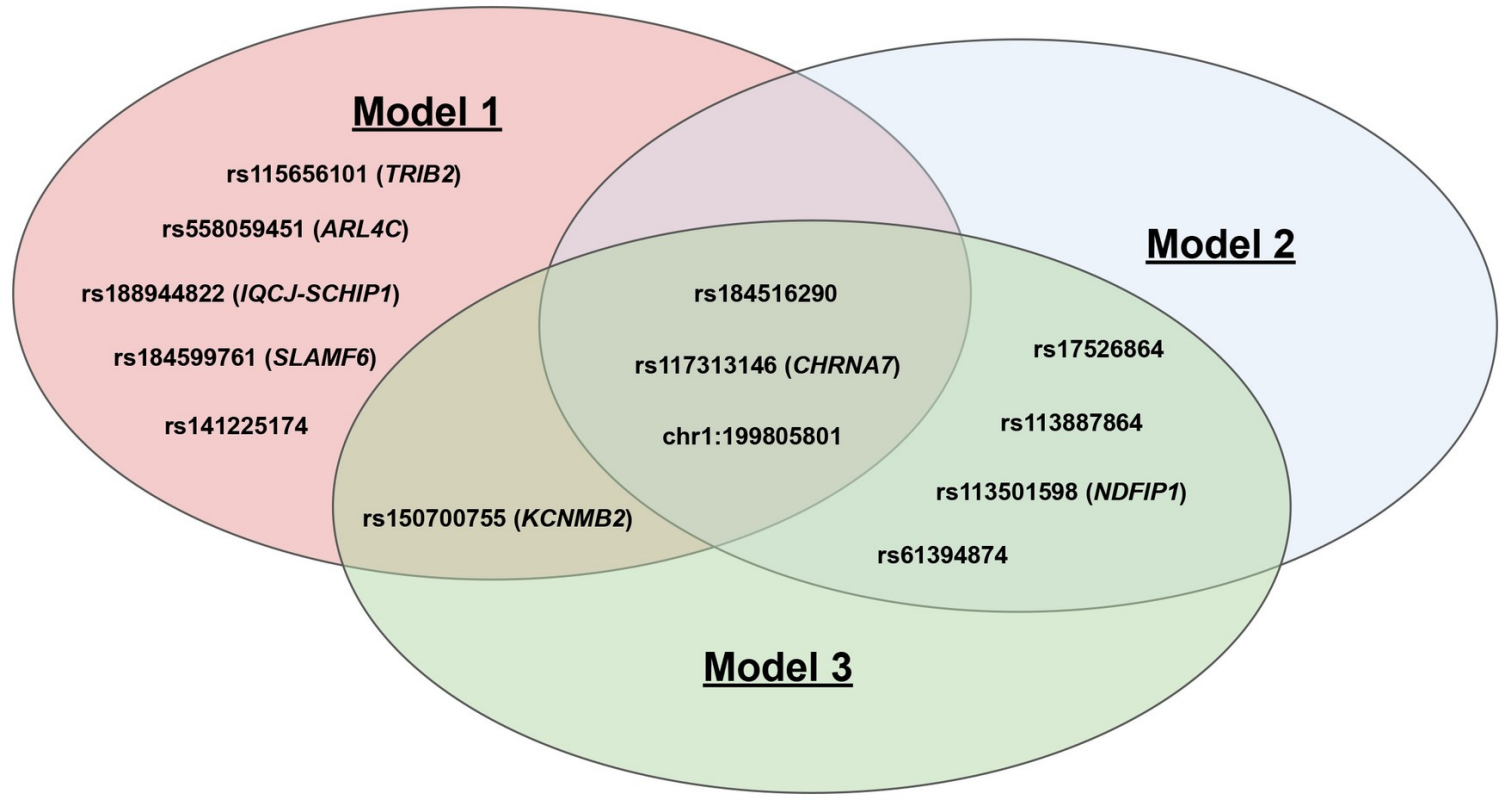
Overlap and Intersection of suggestive variants from the three genome-wide association studies. Gene Names: ARL4C = ADP Ribosylation Factor like GTPase 4C; CHRNA7 = Cholinergic Receptor Nicotinic Alpha 7 Subunit; IQCJ = IQ Domain-Containing Protein J; KCNMB2 = Potassium Calcium-Activated Channel Subfamily M Regulatory Beta Subunit 2; NDFIP1 = Nedd4 Family Interacting Protein 1; SCHIP1 = Schwannomin Interacting Protein 1; SLAMF6 = SLAM Family Member 6; TRIB2 = Tribbles Pseudokinase 2.

Q-Q plots confirmed that the analyses were well-controlled, without an elevated Type I error (S1 Fig). Median genomic control λ values calculated across all tested variants were 1.051, 1.044, and 1.043 for Models 1, 2, and 3, respectively. Median genomic control λ values calculated across only variants with MAF≥ 1% were 1.004, 0.997, and 0.996 for Models 1, 2, and 3 [29]. No variants exceeded our threshold for genome-wide significance (*P*<5x10^-8^), however, a total of 13 variants exceeded the suggestive (*P*<1x10^-6^) threshold in at least one of our Models (Table 2). Manhattan plots for each of the 3 Models can be found in Fig 2. Variants exceeding the suggestive (*P*<1x10^-6^) threshold for each of our 3-models are described in Table 2. Notably, **rs184516290** (chr1:199814965:G:A), chr1:199805801:T:TA, and **rs117313146** (chr15:31999784:G:C) exceeded the suggestive significance threshold in all 3-models. **rs113501598** (chr5:142181517:G:A), **rs17526864** (chr5:142170708:C:T), **rs113887864** (chr5:142179290:G:A), **rs61394874** (chr5:142166870:G:A) exceeded the suggestive threshold in Models 2 and 3 (Table 2). The overlap and intersection of suggestive genetic variants (and accompanying genes) between each of the three genome-wide association studies can be visualized in Fig 3.

**Table 2:** Genome Wide Association Study (GWAS) results: *P* < 1 x 10^−^6, minor allele frequency > 1%.

***A*.** Model 1: GWAS Performed on Primary Outcome (KDIGO Stage 1, 2, or 3)—Controlling for age, sex, chip, and first 5 principal components							
**Chr**	**Pos**	**Test Allele**	**MAF**	**SNP**	**OR**	**SE**	***P*-value**
1	199,814,965	A	0.00122	rs184516290	24,706.2123	1.858	5.22 x 10^−8^
1	199,805,801	TA	0.00120		24,437.7200	1.858	5.36 x 10^−8^
15	31,999,784	C	0.00398	rs117313146	115.1983	0.897	1.21 x 10^−7^
15	30,789,224	G	0.00310	rs141225174	210.6200	1.080	7.27 x 10^−7^
3	178,817,653	C	0.00554	rs150700755	24.2202	0.644	7.43 x 10^−7^
3	159,729,538	C	0.00262	rs188944822	298.2160	1.152	7.52 x 10^−7^
2	234,602,989	A	0.00571	rs558059451	36.0140	0.726	7.95 x 10^−7^
1	160,490,860	A	0.00379	rs184599761	82.2811	0.894	8.00 x 10^−7^
2	12,995,075	C	0.01787	rs115656101	4.9574	0.326	9.35 x 10^−7^
*B*. Model 2: GWAS Performed on Primary Outcome (KDIGO Stage 1, 2, or 3)—Controlling for age, sex, chip, baseline serum creatinine, and first 5 principal components							
**Chr**	**Pos**	**Test Allele**	**MAF**	**SNP**	**OR**	**SE**	***P*-value**
1	199,814,965	A	0.00122	rs184516290	6,989.1694	1.641	6.85 x 10^−8^
1	199,805,801	TA	0.00120		6,936.6793	1.641	7.00 x 10^−8^
5	142,181,517	A	0.04474	rs113501598	3.0326	0.217	3.12 x 10^−7^
5	142,170,708	T	0.04770	rs17526864	2.8538	0.207	4.30 x 10^−7^
5	142,179,290	A	0.04490	rs113887864	2.9647	0.215	4.37 x 10^−7^
5	142,166,870	A	0.04830	rs61394874	2.7563	0.205	7.17 x 10^−7^
1	31,999,784	C	0.00398	rs117313146	67.8608	0.856	8.39 x 10^−7^
***C*.** Model 3: GWAS Performed on Primary Outcome (KDIGO Stage 1, 2, or 3)—Controlling for age, sex, chip, baseline serum creatinine, lactate, total increase in SOFA score, comorbidities: diabetes complicated, diabetes uncomplicated, congestive heart failure, liver disease, peripheral vascular disease, COPD, and cardiac arrhythmias, and first 5 principal components							
**Chr**	**Pos**	**Test Allele**	**MAF**	**SNP**	**OR**	**SE**	***P*-value**
1	199,814,965	A	0.00122	rs184516290	886.1285	1.295	1.60 x 10^−7^
1	199,805,801	TA	0.00120		882.7745	1.295	1.62 x 10^−7^
5	142,181,517	A	0.04474	rs113501598	3.0670	0.219	3.01 x 10^−7^
5	142,179,290	A	0.04490	rs113887864	3.0174	0.217	3.77 x 10^−7^
5	142,170,708	T	0.04770	rs17526864	2.8842	0.209	4.05 x 10^−7^
3	178,817,653	C	0.00554	rs150700755	27.2676	0.657	4.92 x 10^−7^
15	31,999,784	C	0.00398	rs117313146	60.1007	0.820	5.81 x 10^−7^
5	142,166,870	A	0.04830	rs61394874	2.7814	0.206	6.90 x 10^−7^

GWAS results for variants previously reported to be associated with AKI (but not exceeding the suggestive threshold) are also reported to facilitate comparison and replication (S1 Table).

### Sensitivity analysis: Severe AKI

Results of the analysis repeated for severe AKI (defined as KDIGO, Stage 2 or 3) are shown in S2 Table. As with the primary outcome, no variants exceeded the genome-wide significance threshold (*P*<10^−8^), but numerous variants exceeded the suggestive (*P*<1x10^-6^) threshold and appeared across multiple models. Notably, **rs11140550** (chr9:84369833:G:A) and **rs11140551** (chr9:834369848:C:T) near ***SLC28A3*** and **rs2671704** (chr10:48929632:C:T), **rs2377695** (chr10:4829132:G:A), **rs2671703** (chr10:48927949:C:G), **rs2663023** (chr10:48928027:T:C), **rs2663025** (chr10:48928128:G:A), **rs1877803** (chr10:48929468:A:G), and **rs2671706** (chr10:48930286:T:C) near ***WDFY4*** exceeded the suggestive significance threshold in Models 2 and 3.

### Gene expression patterns

We explored the gene expression patterns of the candidate genes suggested by genome-wide association studies using the Genotype-Tissue Expression (GTEx) portal [30]. ***ARL4C*** (kidney-cortex: 7.956 median transcripts per million; kidney-medulla:10.210), ***CHRNA7*** (0.037; 0.016), ***IQCJ-SCHIP1*** (1.111; 1.431), ***NDFIP1*** (46.780; 67.020), ***KCNMB2*** (0.872, 1.861), ***TRIB2*** (29.800; 89.660), and ***SLAMF6*** (0.419; 0.270) were all expressed by the kidneys. Lung was the only tissue that was significantly differentially expressed among our candidate genes on the FUMA test [28] (FDR corrected p-value = 0.0306), driven by candidate genes ***ARL4C***, ***TRIB2***, ***SCHIP1***, and ***SLAMF6***. Kidney, however, was not found to be differentially expressed (FDR corrected p-value = 1).

## Discussion

In a large, unbiased GWAS on S-AKI, we failed to identify any variants reaching genome-wide significance (*P*<5x10^-8^). Numerous loci reached the “suggestive” threshold (*P*<1x10^-6^), [27] however, these should not be viewed as significant novel associations, as adoption of less stringent thresholds leads to increased rate of false discovery [31]. **rs117313146** (chr15:31999784:G:C), near the ***CHRNA7*** gene, was associated with S-AKI at the suggestive threshold in all 3-models presented. While our study is the first to suggest an association between a specific acetylcholine receptor subunit and S-AKI in a human GWAS; nicotinic acetylcholine receptors have been linked to both septic [32] and ischemic [33] AKI in mouse models. Furthermore, the ***CHRNA7*** variant (chr15:32413847:G:A) was associated with the largest odds for developing Sepsis-3 in a recent publication [20].

***CHRNA7*** is the gene for the human *α7 subunit of the neuronal nicotinic acetylcholine receptor gene* and has previously been shown to regulate inflammation by attenuating the production of multiple inflammatory cytokines from macrophages [34, 35]. The *α7* controls systemic inflammation in sepsis by inhibiting TNF production and the NF-kB immunomodulatory response [34]. Inhibition of this pathway has been shown to improve survival in experimental models of sepsis [36]. While our study was likely underpowered to discover new variants at the level of genome-wide significance (*P*<5x10^-8^); the strongly concordant results within our 3-models, confirmation of findings from an overlapping phenotype [20] at replication threshold (*P*<1x10^-6^) [20], and plausible mechanistic link should be viewed as hypothesis-generating. Additional studies are necessary to confirm the association between ***CHRNA7*** and S-AKI as well as identify potential therapeutic interventions that block the inflammatory signaling pathways involving nicotinic acetylcholine receptor α7 subunit.

Additionally, **rs184516290** (chr1:199814965:G:A) and chr1:199805801:T:TA (no rsID) both near the ***NR5A2*** gene exceeded the suggestive threshold in all 3-models. The ***NR5A2*** gene is a pioneer transcription factor which initiates transcription from closed chromatin and regulates metabolism in adult tissues [37]. While not specifically implicated in S-AKI, **NR5A2** regulates the *calreticulin* gene during renal fibrosis [38, 39] and plays a role in multiple immune pathways including inflammation [40] and tumor-necrosis factor- (TNF) induced cell death [41].

All candidate genes were expressed, to varying extent, in the kidneys indicating a potential role in sepsis-associated AKI; however, differential expression within the lung (and not kidney) indicates that the relationship is likely more complex and requires further exploration. Gene expression patterns have been quantified in S-AKI models [42–44]. Notably, the ***ARL4C*** gene (rs558059451; chr2:234602989:G:A, in our study) was downregulated in S-AKI in a previously published Gene Ontology enrichment analysis [42]. Additionally, ***ARL4C***, ***TRIB2***, and ***SLAMF6*** were found to be downregulated within 0.5, 24, and 48-hours after septic shock [44]. Full details on differential expression of suggested genes from our study can be found in S3 Table. Future studies are necessary to assess gene expression patterns of the candidate genes and compare those to published models of sepsis and kidney injury.

The sensitivity analysis restricted to more severe, KDIGO Stage 2 or 3 AKI, cases suggested (*P*<1x10^-6^) a different set of genes (***SLC28A3*** and ***WDFY4***) compared to the primary analysis. This could be due to a different genetic effect between more and less severe AKI, but more likely is because most cases were Stage 1 AKI, leaving the sensitivity analysis of Stage 2 and 3 severely underpowered to detect novel associations.

### Comparison to previously published results

We failed to replicate any of the variants previously reported to be associated with S-AKI at *P*<1x10^-6^ threshold (S1 Table). Variants in the suppressor of fused homolog (***SUFU***) gene previously shown to be correlated with renal function in adult patients hospitalized with *Enterobacteriaceae* bacteremia (at threshold *P*<0.05) [45] also consistently demonstrated *P*<0.05 in our Models (**rs10786691**, *P* = 0.040–0.053; **rs12414407**, *P* = 0.007–0.026; **rs10748825**, *P* = 0.007–0.019; **rs2296590**, *P* = 0.008–0.016) [45]. Although significance-level was consistent between the two studies across multiple ***SUFU*** variants and models, since they did not exceed the *a priori* significance threshold for replication (*P*<1x10^-6^), these findings should be viewed as hypothesis-generating and additional studies are necessary to elucidate the role of the sonic hedgehog signaling pathway in S-AKI.

Inability to replicate findings from other prior studies could be due to differences in how phenotypes were defined. Specifically, we assessed KDIGO Stage 1, 2, or 3 AKI [19] following Sepsis (as defined by the Sepsis-3 diagnostic criteria) [1]. This contrasts with other studies using alternative definitions or severity classifications: (i) RIFLE (AKI) after American College of Chest Physicians/Society of Critical Care Medicine (Sepsis) [46] or AKIN Stage 3 in the setting of severe sepsis or septic shock (based upon SOFA score) [47]. Although our study demonstrated similar significance-levels to prior work from Hanao-Martinez, their outcome was renal function as defined by serum creatinine (continuous),precluding direct effect-size comparisons with our KDIGO AKI outcome (binary) [45]. Alternatively, issues replicating prior studies could be because S-AKI has limited genetic contribution and low heritability (as found in other AKI subtypes) [16, 17]. In this case, a greater understanding of risk for AKI could be gained from characterizing non-genetic clinical risk factors (some of which may be modifiable) rather than from further genetic studies.

In addition to comparing our work to studies on the narrow S-AKI phenotype, we also compare our results to other AKI sub-phenotypes, including: (i) critically ill [18] and (ii) post-surgical populations to facilitate the broadest understanding of overlapping mechanisms [16, 17]. The lack of concordance with these studies could be due to statistical variation in significance between studies or, alternatively, due to unique genetic pathways responsible for S-AKI.

### Study strengths and limitations

Unlike prior studies which have largely employed a candidate-gene approach, [14, 45, 46, 48] our study provides a large, unbiased analysis on the genetics underlying S-AKI. We adjust for multiple clinical covariates (including baseline serum creatinine, lactate prior to diagnosis of sepsis, and SOFA-score). As studies demonstrated considerable variation based upon phenotype definition, [20] our study employs standardized diagnostic criteria for Sepsis (Sepsis-3) [1] and AKI (KDIGO), [19] in a previously validated dataset [13]. We assess for differential genetic influence at greater severity phenotypes in a pre-specified sensitivity analysis. The major limitations of this study are lack of external validation (ie: single-center design) and lack of statistical power to detect novel genetic associations in low-frequency variants or variants with moderate effect sizes.

While our study selected cases and controls using the entire population of the Sepsis Database with available genetic data in Michigan Genomics Initiative, there are potentially unaccounted biases in the initial curation of this dataset. For example, more critically ill patients are likely to have more frequent laboratory draws and vital sign documentation, leading to higher probability of reaching the Sepsis-3 diagnostic criteria. Furthermore, as the data within the data warehouse was generated during the process of providing clinical care (and not specifically for research purposes), additional inter-provider variation and other sources of bias are possible. While we were unable to include stages of AKI based on urine creatinine, this is similar to other studies [13, 49, 50]. Patients with AKI by urine criteria only have better outcomes than patients who meet serum creatinine criteria for AKI [51]. However, our exclusion of urine criteria may bias the results in unknown ways.

While the data within Michigan Genomics Initiative are reflective of the patient population served by Michigan Medicine and lack of ancestral diversity is a commonly encountered problem across many biobanks, [52] lack of genetic diversity (92% European Ancestry) is an inherent limitation of our dataset. Furthermore, analysis of patients at a tertiary academic center in the United States may not reflect findings from international or community centers.

## Conclusions

While failing to identify any novel association for S-AKI at the level of genome-wide significance, our study did suggest multiple variants in previously characterized pathways for S-AKI including ***CHRNA7***, ***NR5A2***, and ***SUFU***. We failed to replicate associations from multiple prior studies which may result from differences in how the phenotype was defined or, alternatively, limited genetic contribution and low heritability.

## Supporting information

S1 Appendix Characterization and quality control within Michigan genomics initiative. (DOCX)

S1 Fig Quantile-Quantile (Q-Q) plots for each of the three genome-wide association studies. (PDF)

S1 Table Attempted replication of prior GWAS findings. * SNP not found in MGI dataset. ** Study by Henao-Martinez *et al*., 2013 used Serum Creatinine (mg/dL) as a marker of renal function (ie: continuous variable), therefore, direct comparison of effect size to our logistic regression, which used KDIGO-staged AKI (ie: binary, categorical variable) is not possible. Studies replicated at *P* < 0.05 threshold are denoted in **bold**. Abbreviations: CHF = congestive heart failure; Chr = Chromosome; COPD = chronic obstructive pulmonary disease; GWAS = genome-wide association study; KDIGO = Kidney Disease: Improving Global Outcomes; MAF = Minor Allele; MGI = Michigan Genomics Initiative OR = Odds Ratio; Pos = Position; Frequency; S-AKI = Sepsis-associated Acute Kidney Injury; SOFA = Sequential Organ Failure Assessment; SE = Standard Error; SNP = single nucleotide polymorphism.
(DOCX)

S2 Table Genome Wide Association Study (GWAS) results: *P* < 1 x 10^−6^, minor allele frequency > 1%. A. Model 1: GWAS Performed on Primary Outcome (KDIGO Stage 2 or 3)—Controlling for age, sex, chip, and first 5 principal components. B. Model 2: GWAS Performed on Primary Outcome (KDIGO Stage 2 or 3)—Controlling for age, sex, chip, baseline serum creatinine, and first 5 principal components. C. Model 3: GWAS Performed on Primary Outcome (KDIGO Stage 2 or 3)—Controlling for age, sex, chip, baseline serum creatinine, lactate, total increase in SOFA score, comorbidities: diabetes complicated/uncomplicated, CHF, liver disease, peripheral vascular disease, COPD, and cardiac arrhythmias, and first 5 principal components. Abbreviations: CHF = congestive heart failure; Chr = Chromosome; COPD = chronic obstructive pulmonary disease; GWAS = genome-wide association study; KDIGO = Kidney Disease: Improving Global Outcomes; MAF = Minor Allele; OR = Odds Ratio; Pos = Position; Frequency; SOFA = Sequential Organ Failure Assessment; SE = Standard Error; SNP = single nucleotide polymorphism.
(DOCX)

S3 Table Differential gene expression of suggested genes in published S-AKI models. Abbreviations: logFC: The log fold change, or the change in gene expression for each unit increase; AveExpr: The average expression across all samples; t: The logFC divided by its standard error; P.Value: The raw p-value based on t from the test that logFC differs from 0.
(DOCX)

## Abbreviations

AKI: Acute Kidney Injury
GWAS: Genome-wide association study
MGI: Michigan Genomics Initiative
MPOG: Multicenter Perioperative Outcomes Group
S-AKI: Sepsis-associated AKI
