# Supplementary material for: Genetic variants associated with sepsis-associated acute kidney injury
Source: PLoS One. 2024 Dec 5;19(12):e0311318. doi: 10.1371/journal.pone.0311318 (PMC11620412; doi:10.1371/journal.pone.0311318)
Supplement: S1 Appendix — (DOCX) [file pone.0311318.s001.docx]

S1 Appendix. Characterization and Quality Control within Michigan Genomics Initiative

The *Michigan Genomics Initiative* excludes SNPs with overall call rate < 99%, high sample missingness (>1%), or significant deviation from Hardy-Weinberg Equilibrium (*P* < 10^-4^) within each array. Principal components (PC) of unrelated individuals were generated using FlashPCA2 v2.0 and individuals with greater than a 3rd degree relative (as determined by KING v2.2.7) were projected onto the resulting PC space.
