# Supplementary figures and images for: Genetic variants associated with sepsis-associated acute kidney injury
Source: PLoS One. 2024 Dec 5;19(12):e0311318. doi: 10.1371/journal.pone.0311318 (PMC11620412; doi:10.1371/journal.pone.0311318)

**Model 1**

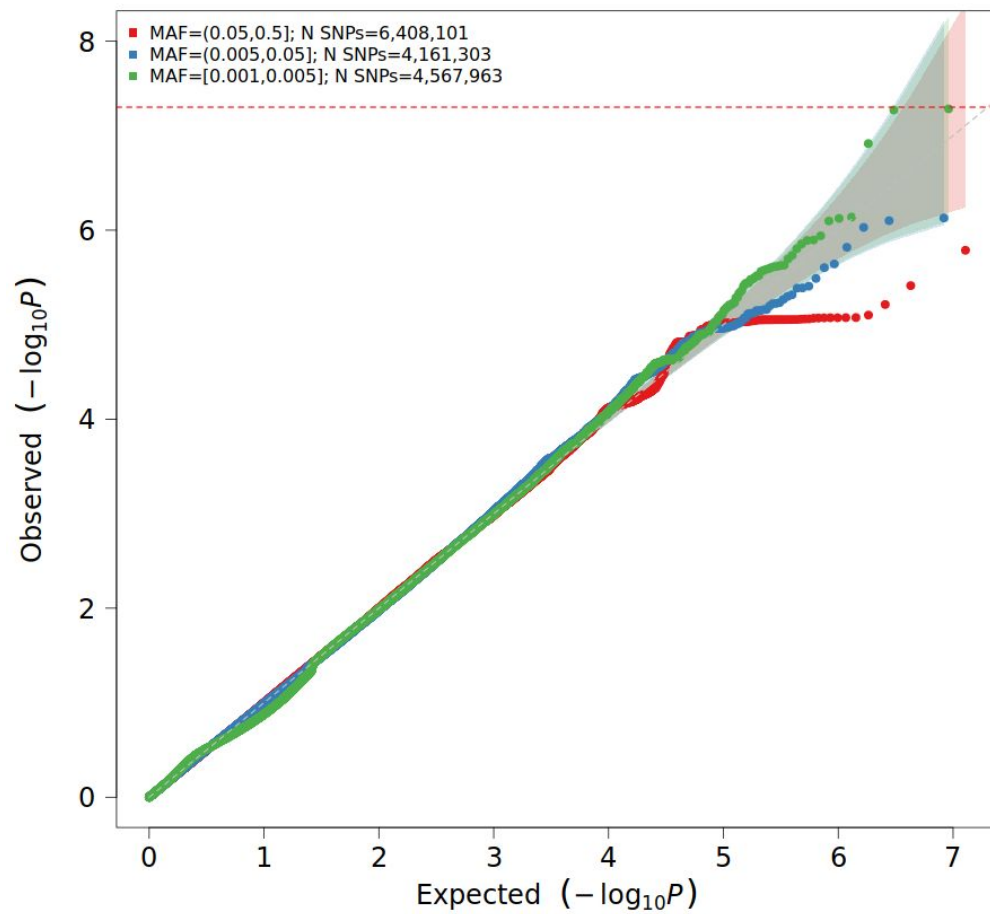

**Model 2**

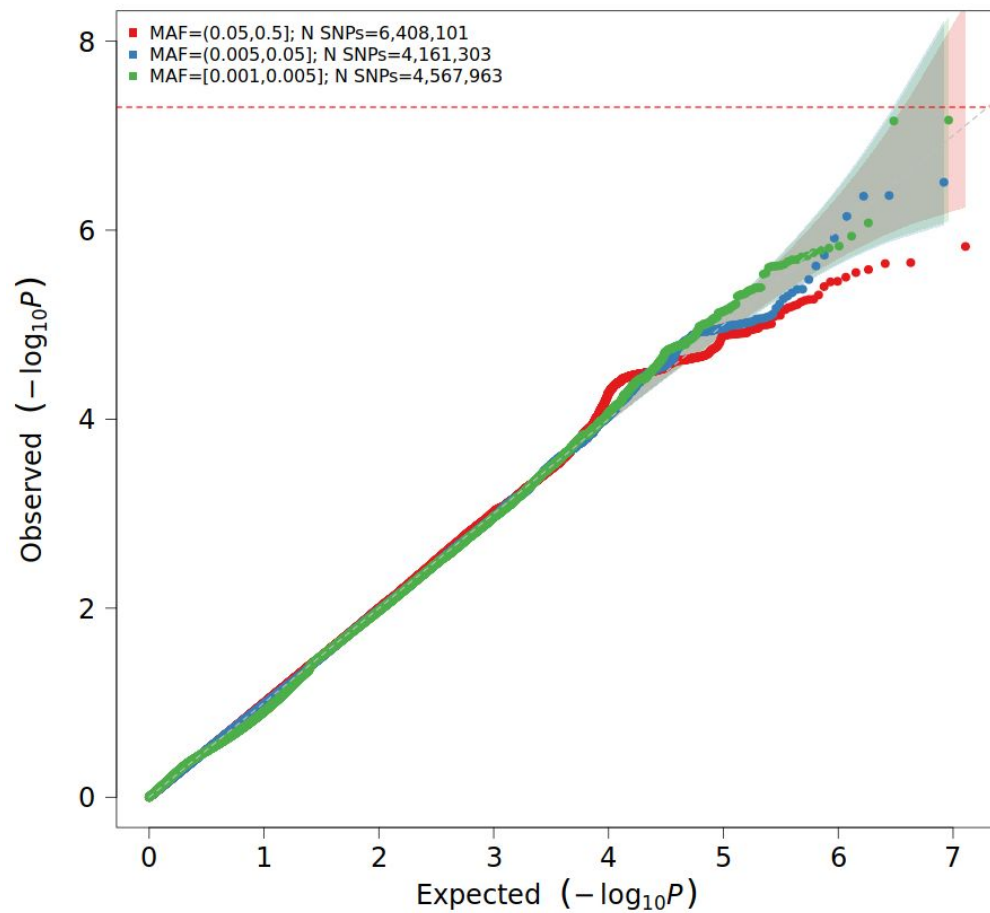

**Model 3**

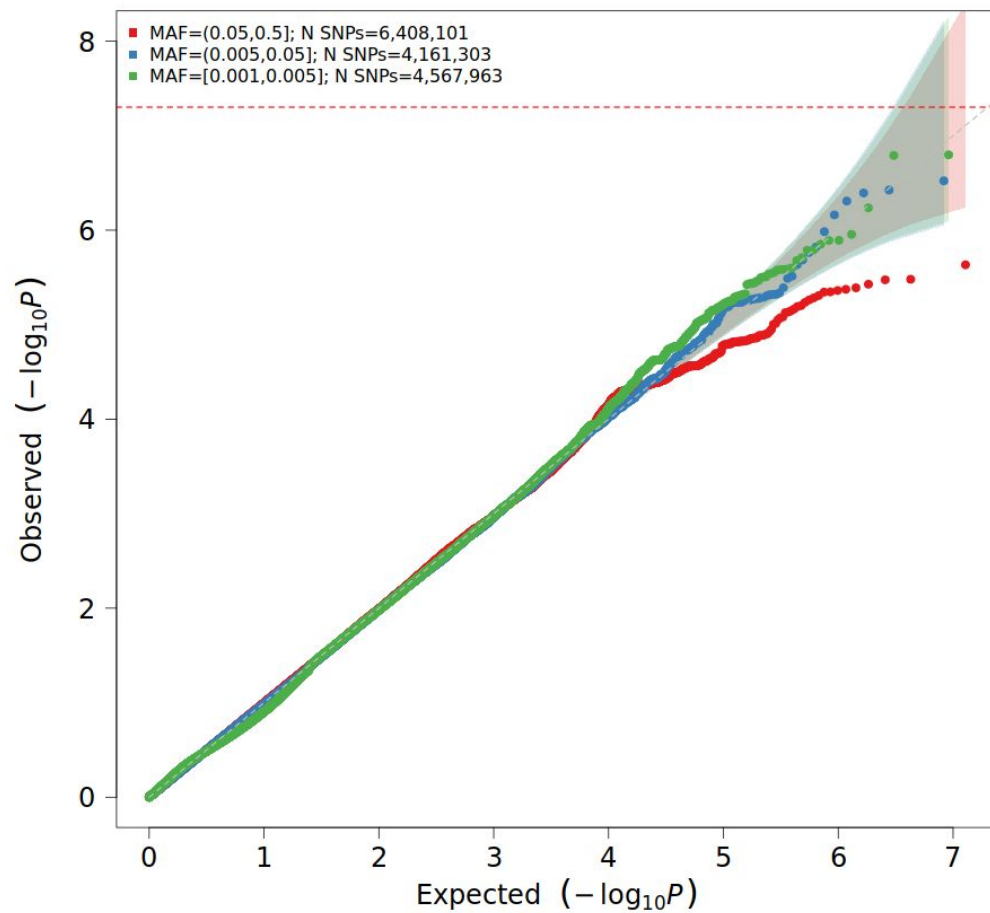

Supplement: S1 Fig — (PDF) [file pone.0311318.s002.pdf]
