# Supplementary material for: Genetic variants associated with sepsis-associated acute kidney injury
Source: PLoS One. 2024 Dec 5;19(12):e0311318. doi: 10.1371/journal.pone.0311318 (PMC11620412; doi:10.1371/journal.pone.0311318)
Supplement: S1 Table — * SNP not found in MGI dataset. ** Study by Henao-Martinez et al., 2013 used Serum Creatinine (mg/dL) as a marker of renal function (ie: continuous variable), therefore, direct comparison of effect size to our logistic regression, which used KDIGO-staged AKI (ie: binary, categorical variable) is not possible. Studies replicated at P < 0.05 threshold are denoted in bold. Abbreviations: CHF = congestive heart failure; Chr = Chromosome; COPD = chronic obstructive pulmonary disease; GWAS = genome-wide association study; KDIGO = Kidney Disease: Improving Global Outcomes; MAF = Minor Allele; MGI = Michigan Genomics Initiative OR = Odds Ratio; Pos = Position; Frequency; S-AKI = Sepsis-associated Acute Kidney Injury; SOFA = Sequential Organ Failure Assessment; SE = Standard Error; SNP = single nucleotide polymorphism. (DOCX) [file pone.0311318.s003.docx]

**S3 Table. Attempted Replication of Prior GWAS findings**

|  | | *Discovery cohort*  Cardinal-Fernandez [*et al*., 201](https://www.ncbi.nlm.nih.gov/pmc/articles/PMC4589439/)3 | | | *Replication Cohort*  S-AKI/MGI, 2024 | | |
| --- | --- | --- | --- | --- | --- | --- | --- |
| **SNPID** | **Gene** | **MAF** | **OR** | **P-value** | **MAF** | **OR** | **P-value** |
| rs1800750 | TNFα (-376) | 0.060 | 1.01 (0.37-2.80) | 0.98 | 0.012 | 1.19 (0.59-2.39) | 0.632 |
|  |  |  |  |  | 0.012 | 1.22 (0.59-2.50) | 0.596 |
|  |  |  |  |  | 0.012 | 1.29 (0.61-2.73) | 0.505 |
| rs1800629 | TNFα (-308) | 0.013 | 0.81 (0.37-1.75) | 0.59 | 0.174 | 1.07 (0.88-1.31) | 0.482 |
|  |  |  |  |  | 0.174 | 1.07 (0.86-1.33) | 0.527 |
|  |  |  |  |  | 0.174 | 1.07 (0.86-1.34) | 0.518 |
| rs361525 | TNFα (-238) | 0.040 | 1.67 (0.50-5.53) | 0.41 | 0.053 | 0.79 (0.56-1.11) | 0.171 |
|  |  |  |  |  | 0.053 | 0.87 (0.60-1.27) | 0.469 |
|  |  |  |  |  | 0.053 | 0.88 (0.60-1.29) | 0.515 |
| rs4073 | IL-8 (-251) | 0.430 | 4.98 (1.04-23.86) | 0.09 | 0.519 | 1.00 (0.86-1.17) | 0.961 |
|  |  |  |  |  | 0.519 | 1.03 (0.87-1.22) | 0.699 |
|  |  |  |  |  | 0.519 | 1.01 (0.85-1.19) | 0.932 |
| rs970242 | PBEF (-1001) | 0.230 | * | <0.01 | * | * | * |
| rs3025039 | VEGF (+936) | 0.110 | 2.64 (1.07-6.49) | 0.03 | 0.145 | 1.11 (0.88-1.41) | 0.376 |
|  |  |  |  |  | 0.145 | 1.11 (0.86-1.43) | 0.435 |
|  |  |  |  |  | 0.145 | 1.12 (0.86-1.44) | 0.406 |
| rs2010963 | VEGF (+405) | 0.380 | 1.33 (0.48-3.70) | 0.58 | 0.669 | 0.96 (0.81-1.15) | 0.681 |
|  |  |  |  |  | 0.669 | 0.99 (0.82-1.20) | 0.926 |
|  |  |  |  |  | 0.669 | 0.97 (0.80-1.17) | 0.745 |
| rs4646994 | ACEII | 0.420 | 1.15 (0.48-2.74) | 0.76 | * | * | * |
|  | | Henao-Martinez *et al*., 2013 ** | | | *Replication Cohort*  S-AKI/MGI, 2024 | | |
| rs10786691 | SUFU |  | -0.16  (-0.30- -0.02) | 0.03 | 0.416 | 1.17 (1.00-1.36) | 0.053 |
|  |  |  |  |  | **0.416** | **1.19 (1.01-1.41)** | **0.042** |
|  |  |  |  |  | **0.416** | **1.19 (1.01-1.41)** | **0.040** |
| rs12414407 | SUFU |  | -0.18  (-0.33 - -0.02) | 0.026 | **0.660** | **0.83 (0.70-0.98)** | **0.026** |
|  |  |  |  |  | **0.660** | **0.79 (0.66-0.94)** | **0.009** |
|  |  |  |  |  | **0.660** | **0.78 (0.65-0.94)** | **0.007** |
| rs10748825 | SUFU |  | -0.19  (-0.34 - -0.05) | 0.01 | **0.329** | **1.22 (1.03-1.44)** | **0.019** |
|  |  |  |  |  | **0.329** | **1.28 (1.07-1.53)** | **0.008** |
|  |  |  |  |  | **0.329** | **1.28 (1.07-1.53)** | **0.007** |
| rs10748827 | SUFU |  | -0.14  (-0.30 - 0.006) | 0.06 | **0.327** | **1.22 (1.04-1.44)** | **0.018** |
|  |  |  |  |  | **0.327** | **1.28 (1.07-1.53)** | **0.008** |
|  |  |  |  |  | **0.327** | **1.28 (1.07-1.54)** | **0.007** |
| s7078511 | SUFU |  | -0.20  (-0.34- -0.06) | 0.006 | 0.548 | 0.89 (0.76-1.04) | 0.144 |
|  |  |  |  |  | 0.548 | 0.87 (0.74-1.03) | 0.110 |
|  |  |  |  |  | 0.548 | 0.87 (0.74-1.03) | 0.112 |
| rs2296590 | SUFU | 0.325 | -0.23  (-0.39- -0.76) | 0.049 | **0.335** | **1.23 (1.04-1.45)** | **0.016** |
|  |  |  |  |  | **0.335** | **1.27 (1.06-1.52)** | **0.008** |
|  |  |  |  |  | **0.335** | **1.28 (1.06-1.53)** | **0.008** |
|  |  | Zhao *et al*., 2017 | | | *Replication Cohort*  S-AKI/MGI, 2024 | | |
| rs62341639 | APOL1 regulator, IRF2 | 0.18/0.14 | 0.64 | 2.48x10^-7^ | 0.202 | 0.98 (0.80-1.20) | 0.854 |
|  |  |  |  |  | 0.202 | 0.97 (0.78-1.20) | 0.762 |
|  |  |  |  |  | 0.202 | 0.96 (0.77-1.19) | 0.711 |
| rs62341657 | APOL1 regulator, IRF2 | 0.18/0.14 | 0.65 | 3.26x10^-7^ | 0.200 | 0.97 (0.80-1.19) | 0.789 |
|  |  |  |  |  | 0.200 | 0.96 (0.78-1.19) | 0.711 |
|  |  |  |  |  | 0.200 | 0.95 (0.77-1.18) | 0.657 |
| rs9617814 | AKI-related gene TBX1 | 0.22/  0.20 | 0.70 | 3.81x10^-6^ | 0.251 | 0.93 (0.78-1.11) | 0.416 |
|  |  |  |  |  | 0.251 | 0.96 (0.80-1.16) | 0.660 |
|  |  |  |  |  | 0.251 | 0.93 (0.77-1.13) | 0.475 |
| rs10854554 | AKI-related gene TBX1 | 0.19/  0.17 | 0.67 | 6.53x10^-7^ | 0.218 | 0.98 (0.82-1.18) | 0.853 |
|  |  |  |  |  | 0.218 | 1.01 (0.83-1.24) | 0.887 |
|  |  |  |  |  | 0.218 | 0.99 (0.81-1.21) | 0.931 |
|  | | Frank *et al*., 2012 | | |  | | |
| rs625145 | SIK3 | 0.26/  0.19 | 1.64 (1.19-2.28) | 0.0028 | 0.209 | 0.94 (0.78-1.13) | 0.514 |
|  |  |  |  |  | 0.209 | 0.97 (0.79-1.20) | 0.805 |
|  |  |  |  |  | 0.209 | 0.97 (0.79-1.19) | 0.755 |
| rs2093266 | SERPINA4 | 0.07/  0.17 | 0.53 (0.34-0.82) | 0.0042 | 0.105 | 1.21 (0.95-1.55) | 0.122 |
|  |  |  |  |  | 0.105 | 1.24 (0.95-1.62) | 0.111 |
|  |  |  |  |  | 0.105 | 1.24 (0.95-1.63) | 0.113 |
| rs1955656 | SERPINA5 | 0.07/  0.13 | 0.52 (0.33-0.81) | 0.0037 | 0.105 | 1.21 (0.95-1.55) | 0.123 |
|  |  |  |  |  | 0.105 | 1.24 (0.95-1.61) | 0.114 |
|  |  |  |  |  | 0.105 | 1.24 (0.95-1.62) | 0.117 |
| rs8094315 | BCL2 | 0.17/  0.25 | 0.62 (0.45-0.85) | 0.0032 | 0.215 | 1.06 (0.87-1.28) | 0.562 |
|  |  |  |  |  | 0.215 | 0.98 (0.80-1.20) | 0.845 |
|  |  |  |  |  | 0.215 | 0.98 (0.80-1.20) | 0.849 |
| rs12457893 | BCL2 | 0.42/  0.51 | 0.68 (0.52-0.88) | 0.0034 | 0.451 | 1.03 (0.89-1.21) | 0.680 |
|  |  |  |  |  | 0.451 | 0.98 (0.83-1.16) | 0.836 |
|  |  |  |  |  | 0.451 | 1.00 (0.84-1.18) | 0.996 |
|  |  | Vilander *et al.*, 2019 | | |  | | |
| rs1800629 | TNFA | 0.15  /0.14 | 1.06 (0.89-1.28) | 0.51 | 0.174 | 1.07 (0.88-1.31) | 0.482 |
|  |  |  |  |  | 0.174 | 1.07 (0.86-1.33) | 0.527 |
|  |  |  |  |  | 0.174 | 1.07 (0.86-1.34) | 0.518 |
| rs1800896 | IL10 | 0.44/  0.46 | 0.92 (0.80-1.05) | 0.20 | 0.473 | 0.96 (0.83-1.12) | 0.643 |
|  |  |  |  |  | 0.473 | 0.97 (0.83-1.14) | 0.731 |
|  |  |  |  |  | 0.473 | 0.96 (0.82-1.13) | 0.649 |
| rs10499563 | IL6 | 0.15/  0/14 | 1.07 (0.90-1.28) | 0.45 | 0.218 | 1.01 (0.84-1.21) | 0.927 |
|  |  |  |  |  | 0.218 | 1.04 (0.86-1.27) | 0.678 |
|  |  |  |  |  | 0.218 | 1.04 (0.85-1.27) | 0.712 |
| rs1800796 | IL6 | 0.03/  0.03 | 0.88 (0.60-1.29) | 0.51 | 0.049 | 0.82 (0.58-1.16) | 0.273 |
|  |  |  |  |  | 0.049 | 0.70 (0.48-1.03) | 0.069 |
|  |  |  |  |  | 0.049 | 0.72 (0.49-1.06) | 0.100 |
| rs1800795 | IL6 | 0.47  /0.47 | 1.00  (0.88-1.14) | 0.97 | 0.557 | 0.92 (0.78-1.07) | 0.260 |
|  |  |  |  |  | 0.557 | 0.90 (0.76-1.06) | 0.204 |
|  |  |  |  |  | 0.557 | 0.91 (0.77-1.08) | 0.267 |
| rs1474347 | IL6 | 0.47/  0.47 | 1.00 (0.88-1.14) | 1.00 | 0.558 | 0.93 (0.80-1.08) | 0.346 |
|  |  |  |  |  | 0.558 | 0.92 (0.78-1.08) | 0.307 |
|  |  |  |  |  | 0.558 | 0.93 (0.78-1.10) | 0.383 |
| rs13306435 | IL6 | 0.03/  0.03 | 0.91 (0.62-1.33) | 0.62 | 0.010 | 0.66 (0.31-1.39) | 0.274 |
|  |  |  |  |  | 0.010 | 0.77 (0.34-1.76) | 0.536 |
|  |  |  |  |  | 0.010 | 0.81 (0.34-1.91) | 0.633 |
| rs4073 | CXCL8 | 0.42/  0.42 | 0.93  (0.82–1.070 | 0.31 | 0.519 | 1.00 (0.86-1.17) | 0.961 |
|  |  |  |  |  | 0.519 | 1.03 (0.87-1.22) | 0.699 |
|  |  |  |  |  | 0.519 | 1.01 (0.85-1.19) | 0.932 |
| rs2070744 | NOS3 | 0.34/  0.36 | 0.94  (0.82–1.08) | 0.37 | 0.614 | 1.07 (0.92-1.26) | 0.361 |
|  |  |  |  |  | 0.614 | 1.02 (0.86-1.20) | 0.859 |
|  |  |  |  |  | 0.614 | 1.02 (0.86-1.21) | 0.844 |
| rs1050851 | NFKB1A | 0.16/  0.17 | 0.95  (0.79–1.13) | 0.54 | 0.224 | 1.00 (0.83-1.20) | 0.966 |
|  |  |  |  |  | 0.224 | 1.01 (0.83-1.24) | 0.902 |
|  |  |  |  |  | 0.224 | 1.02 (0.83-1.25) | 0.853 |
| rs699 | AGT | 0.43/  0.42 | 1.03  (0.85–1.24) | 0.78 | 0.407 | 1.03 (0.88-1.20) | 0.720 |
|  |  |  |  |  | 0.407 | 1.06 (0.89-1.25) | 0.511 |
|  |  |  |  |  | 0.407 | 1.07 (0.91-1.27) | 0.409 |
| rs2493133 | AGT | 0.41/  0.42 | 0.94  (0.82–1.07) | 0.36 | 0.405 | 1.02 (0.87-1.19) | 0.812 |
|  |  |  |  |  | 0.405 | 1.05 (0.88-1.24) | 0.588 |
|  |  |  |  |  | 0.405 | 1.06 (0.90-1.26) | 0.490 |
| rs2010963 | VEGFA | 0.22/  0.24 | 0.91  (0.78–1.06) | 0.22 | 0.669 | 0.96 (0.81-1.15) | 0.681 |
|  |  |  |  |  | 0.669 | 0.99 (0.82-1.20) | 0.926 |
|  |  |  |  |  | 0.669 | 0.97 (0.80-1.17) | 0.745 |
| rs3025039 | VEGFA | 0.16/  0.14 | 1.20  (1.01–1.44) | 0.044 | 0.145 | 1.11 (0.88-1.41) | 0.376 |
|  |  |  |  |  | 0.145 | 1.11 (0.86-1.43) | 0.435 |
|  |  |  |  |  | 0.145 | 1.12 (0.86-1.44) | 0.406 |
| rs1617640 | EPO | 0.44/  0.45 | 0.99  (0.87–1.13) | 0.91 | 0.591 | 0.98 (0.84-1.14) | 0.776 |
|  |  |  |  |  | 0.591 | 0.93 (0.79-1.10) | 0.393 |
|  |  |  |  |  | 0.591 | 0.95 (0.80-1.12) | 0.542 |
| rs10748825 | SUFU | 0.37/  0.37 | 1.02  (0.88–1.17) | 0.83 | 0.329 | 1.22 (1.03-1.44) | 0.019 |
|  |  |  |  |  | 0.329 | 1.28 (1.07-1.53) | 0.008 |
|  |  |  |  |  | 0.329 | 1.28 (1.07-1.53) | 0.007 |
| rs11549465 | HIF1A | 0.05/  0.04 | 1.19  (0.87–1.62) | 0.28 | 0.092 | 0.89 (0.69-1.16) | 0.386 |
|  |  |  |  |  | 0.092 | 0.97 (0.73-1.29) | 0.847 |
|  |  |  |  |  | 0.092 | 0.97 (0.73-1.30) | 0.861 |
| rs876493 | PNMT | 0.36/  0.38 | 0.94  (0.82–1.08) | 0.37 | 0.554 | 1.09 (0.93-1.27) | 0.274 |
|  |  |  |  |  | 0.554 | 1.09 (0.92-1.28) | 0.342 |
|  |  |  |  |  | 0.554 | 1.10 (0.93-1.30) | 0.281 |
| rs7208693 | MPO | 0.10/  0.12 | 0.88  (0.71–1.09) | 0.23 | 0.073 | 1.01 (0.76-1.36) | 0.925 |
|  |  |  |  |  | 0.073 | 1.04 (0.75-1.43) | 0.814 |
|  |  |  |  |  | 0.073 | 1.03 (0.74-1.42) | 0.865 |
| rs4680 | COMT | 0.47/  0.45 | 1.06  (0.93–1.21) | 0.39 | 0.518 | 1.02 (0.88-1.19) | 0.792 |
|  |  |  |  |  | 0.518 | 0.98 (0.83-1.15) | 0.761 |
|  |  |  |  |  | 0.518 | 0.99 (0.84-1.17) | 0.936 |
| rs2868371 | HSPB1 | 0.19/  0.21 | 0.89  (0.76–1.05) | 0.18 | 0.249 | 0.91 (0.76-1.09) | 0.292 |
|  |  |  |  |  | 0.249 | 0.93 (0.77-1.13) | 0.456 |
|  |  |  |  |  | 0.249 | 0.94 (0.77-1.14) | 0.519 |
| rs2243639 | SFTPD | 0.39/  0.40 | 0.95  (0.83–1.09) | 0.47 | 0.593 | 0.96 (0.83-1.13) | 0.646 |
|  |  |  |  |  | 0.593 | 1.02 (0.87-1.21) | 0.772 |
|  |  |  |  |  | 0.593 | 1.02 (0.86-1.21) | 0.844 |
| rs721917 | SFTPD | 0.39/  0.39 | 1.00  (0.88–1.15) | 0.97 | 0.419 | 1.07 (0.91-1.25) | 0.413 |
|  |  |  |  |  | 0.419 | 1.09 (0.92-1.29) | 0.311 |
|  |  |  |  |  | 0.419 | 1.08 (0.91-1.28) | 0.386 |
| rs10421768 | HAMP | 0.23/  0.25 | 0.88  (0.75–1.03) | 0.11 | 0.232 | 0.95 (0.80-1.14) | 0.594 |
|  |  |  |  |  | 0.232 | 0.95 (0.78-1.15) | 0.587 |
|  |  |  |  |  | 0.232 | 0.92 (0.76-1.12) | 0.434 |
| rs10262995 | BBS9 | 0.04/  0.04 | 0.98  (0.71–1.38) | 0.93 | 0.104 | 1.08 (0.84-1.39) | 0.555 |
|  |  |  |  |  | 0.104 | 1.11 (0.85-1.46) | 0.436 |
|  |  |  |  |  | 0.104 | 1.07 (0.82-1.41) | 0.614 |
|  |  | Bhatraju *et al*., 2023 | | |  | | |
| rs17538288 | DISP1-TLR5 | 0.439 | 1.54  (1.31-1.81) | 1.47×10^-7^ | 0.443 | 1.00 (0.85-1.17) | 0.964 |
|  |  |  |  |  | 0.443 | 1.00 (0.84-1.19) | 0.995 |
|  |  |  |  |  | 0.443 | 1.01 (0.85-1.21) | 0.870 |
| rs7546189 | DISP1-TLR5 | 0.347 | 1.54  (1.3-1.82) | 4.85×10^-7^ | 0.346 | 0.98 (0.83-1.15) | 0.772 |
|  |  |  |  |  | 0.346 | 0.97 (0.81-1.16) | 0.744 |
|  |  |  |  |  | 0.346 | 0.98 (0.82-1.18) | 0.841 |
| **rs80052123** | **GBP3 (5’)** | **0.143** | **1.71**  **(1.36-2.15)** | **3.41×10^-6^** | **0.170** | **0.79 (0.64-0.98)** | **0.031** |
|  |  |  |  |  | **0.170** | **0.79 (0.63-0.99)** | **0.043** |
|  |  |  |  |  | **0.170** | **0.80 (0.63-1.00)** | **0.053** |
| rs6533107 | TACR3 5’ | 0.352 | 0.66  (0.56-0.78) | 1.09×10^-6^ | 0.609 | 1.17 (1.00-1.38) | 0.048 |
|  |  |  |  |  | 0.609 | 1.16 (0.97-1.38) | 0.098 |
|  |  |  |  |  | 0.609 | 1.18 (0.99-1.41) | 0.064 |
| rs9998646 | LINC02492 | 0.441 | 0.68  (0.59-0.80) | 1.22×10^-6^ | 0.560 | 1.04 (0.89-1.22) | 0.600 |
|  |  |  |  |  | 0.560 | 1.07 (0.90-1.27) | 0.437 |
|  |  |  |  |  | 0.560 | 1.07 (0.90-1.27) | 0.421 |
| rs72607731 | LOC101928283 | 0.237 | 1.59  (1.32-1.92) | 1.65×10^-6^ | 0.261 | 1.06 (0.88-1.26) | 0.551 |
|  |  |  |  |  | 0.261 | 1.08 (0.90-1.31) | 0.406 |
|  |  |  |  |  | 0.261 | 1.08 (0.89-1.31) | 0.445 |
| rs1368999 | LINGO2 | 0.492 | 0.67  (0.57-0.80) | 3.32×10^-6^ | 0.529 | 1.10 (0.94-1.28) | 0.249 |
|  |  |  |  |  | 0.529 | 1.11 (0.94-1.31) | 0.232 |
|  |  |  |  |  | 0.529 | 1.13 (0.95-1.33) | 0.171 |
| rs4414368 | Gene Desert | 0.178 | 0.58  (0.46-0.72) | 1.64×10^-6^ | 0.865 | 1.12 (0.89-1.41) | 0.342 |
|  |  |  |  |  | 0.865 | 1.14 (0.89-1.46) | 0.308 |
|  |  |  |  |  | 0.865 | 1.19 (0.92-1.52) | 0.182 |
| rs9945894 | NDUFV2 59 | 0.077 | 2.03  (1.50-2.75) | 4.83×10^-6^ | 0.073 | 1.23 (0.91-1.66) | 0.182 |
|  |  |  |  |  | 0.073 | 1.41 (1.02-1.95) | 0.038 |
|  |  |  |  |  | 0.073 | 1.40 (1.01-1.94) | 0.046 |
|  |  | Larach *et al*., 2022 | | |  | | |
| rs975593 | D21S2088E \| LINC01689 | BioVU: 0.11/0.15  MGI: 0.12/0.15 | 1.38 (1.20–1.59)  1.28 (1.01–1.62) | 6.62×10^-7^ | 0.123 | 1.05 (0.83-1.33) | 0.659 |
|  |  |  |  |  | 0.123 | 1.07 (0.83-1.37) | 0.616 |
|  |  |  |  |  | 0.123 | 1.08 (0.84-1.40) | 0.555 |
| rs2255595 | ADARB2 | BioVU: 0.57/0.53  MGI: 0.59/0.53 | 0.81 (0.73–0.89)  0.80 (0.68–0.93) | 8.62×10^-7^ | 0.433 | 1.13 (0.96-1.33) | 0.138 |
|  |  |  |  |  | 0.433 | 1.11 (0.93-1.32) | 0.232 |
|  |  |  |  |  | 0.433 | 1.12 (0.94-1.33) | 0.198 |
| rs143469518 | PRR15L \| CDK5RAP3 | BioVU: 0.02/0.04  MGI: 0.03/0.04 | 1.88 (1.46–2.44)  1.43 (0.88–2.30) | 8.46×10^-7^ | 0.029 | 1.33 (0.85-2.08) | 0.219 |
|  |  |  |  |  | 0.029 | 1.30 (0.80-2.12) | 0.291 |
|  |  |  |  |  | 0.029 | 1.34 (0.81-2.20) | 0.251 |
| rs2069295 | SP2-AS1 | BioVU: 0.02/0.04  MGI: 0.03/0.04 | 0.49 (0.37–0.65)  0.67 (0.39–1.14) | 4.84×10^-7^ | 0.030 | 1.31 (0.84-2.05) | 0.238 |
|  |  |  |  |  | 0.030 | 1.29 (0.80-2.10) | 0.297 |
|  |  |  |  |  | 0.030 | 1.33 (0.81-2.18) | 0.257 |
| rs117284771 | CDK5RAP3 | BioVU: 0.02/0.04  MGI: 0.03/0.04 | 0.48 (0.36–0.63)  0.74 (0.43–1.25) | 3.69×10^-7^ | 0.030 | 1.30 (0.83-2.04) | 0.247 |
|  |  |  |  |  | 0.030 | 1.28 (0.79-2.09) | 0.315 |
|  |  |  |  |  | 0.030 | 1.32 (0.80-2.16) | 0.273 |
|  |  | Douville *et al*., 2023 | | |  | | |
| rs12421245 | ZNF215 | BioVU: 0.10/0.16  MGI: 0.13/0.21 | 1.80 (1.35–2.40)  1.99 (1.31–3.06) | 3.86×10^-7^ | 0.119 | 1.03 (0.80-1.32) | 0.825 |
|  |  |  |  |  | 0.119 | 1.05 (0.80-1.37) | 0.729 |
|  |  |  |  |  | 0.119 | 1.03 (0.79-1.35) | 0.831 |
| rs73131342 | CDH26 | BioVU: 0.02/0.06  MGI: 0.02/0.06 | 3.30 (1.86–5.83)  3.05 (1.37–6.79) | 8.76×10^-7^ | 0.032 | 1.32 (0.81-2.14) | 0.271 |
|  |  |  |  |  | 0.032 | 1.20 (0.71-2.02) | 0.492 |
|  |  |  |  |  | 0.032 | 1.19 (0.71-2.02) | 0.510 |
| rs3847598 | LMO2 \| CAPRIN1 | BioVU: 0.18/0.24  MGI: 0.16/0.26 | 0.63 (0.50–0.80)  0.52 (0.35–0.78) | 7.55×10^-7^ | 0.779 | 0.98 (0.79-1.21) | 0.824 |
|  |  |  |  |  | 0.779 | 1.03 (0.82-1.30) | 0.787 |
|  |  |  |  |  | 0.779 | 1.05 (0.83-1.32) | 0.694 |
| rs113741905 | RCC2 \| ARHGEF10L | BioVU: 0.20/0.15  MGI: 0.25/0.13 | 0.57 (0.41–0.78)  0.44 (0.30–0.65) | 1.47×10^-7^ | 0.205 | 0.96 (0.79-1.17) | 0.713 |
|  |  |  |  |  | 0.205 | 0.95 (0.77-1.17) | 0.635 |
|  |  |  |  |  | 0.205 | 0.95 (0.76-1.17) | 0.612 |
| rs74637005 | NFU1 | BioVU: 0.03/0.06  MGI: 0.02/0.05 | 3.26 (1.89–5.63)  3.35 (1.27–8.86) | 9.58×10^-7^ | 0.044 | 1.00 (0.67-1.49) | 0.986 |
|  |  |  |  |  | 0.044 | 1.01 (0.66-1.57) | 0.954 |
|  |  |  |  |  | 0.044 | 0.98 (0.63-1.51) | 0.915 |
| **rs17438465** | **EVX1 \| HIBADH** | **BioVU: 0.40/0.30**  **MGI: 0.40/0.31** | **0.60 (0.47–0.75)**  **0.67 (0.48–0.93)** | **8.74×10^-7^** | **0.376** | **1.22 (1.05-1.43)** | **0.012** |
|  |  |  |  |  | **0.376** | **1.28 (1.08-1.52)** | **0.004** |
|  |  |  |  |  | **0.376** | **1.29 (1.09-1.53)** | **0.003** |
|  |  | Stafford-Smith *et al*., 2016 | | |  | | |
| rs1488349 | GRM7, LMCD1 | 0.02/0.01 | 29.71 | 5.41×10^−10^ | 0.975 | 1.34 (0.75-2.39) | 0.323 |
|  |  |  |  |  | 0.975 | 1.31 (0.70-2.45) | 0.392 |
|  |  |  |  |  | 0.975 | 1.30 (0.69-2.45) | 0.419 |
| rs28619003 | BBS9 | 0.08/0.10 | 15.58 | 6.51×10^−8^ | 0.102 | 1.04 (0.81-1.34) | 0.761 |
|  |  |  |  |  | 0.102 | 1.07 (0.81-1.41) | 0.637 |
|  |  |  |  |  | 0.102 | 1.03 (0.78-1.36) | 0.839 |
| rs13317787 | GRM7\|LMCD1-AS1 | 0.03/0.02 | 21.56 | 5.35×10^−7^ | 0.023 | 0.67 (0.41-1.12) | 0.130 |
|  |  |  |  |  | 0.023 | 0.70 (0.40-1.23) | 0.220 |
|  |  |  |  |  | 0.023 | 0.72 (0.41-1.26) | 0.248 |
| rs10262995 | BBS9 | 0.09/0.10 | 14.33 | 2.24×10^−7^ | 0.104 | 1.08 (0.84-1.39) | 0.555 |
|  |  |  |  |  | 0.104 | 1.11 (0.85-1.46) | 0.436 |
|  |  |  |  |  | 0.104 | 1.07 (0.82-1.41) | 0.614 |
|  |  | [Westphal *et al*., 2019](https://pubmed.ncbi.nlm.nih.gov/30678657/) | | |  | | |
| [rs78064607](https://www.ebi.ac.uk/gwas/variants/rs78064607) | [PHLPP2](https://www.ebi.ac.uk/gwas/genes/PHLPP2) | 0.01 | 50 | 3.77x10^-8^ | 0.004 | 0.91 (0.26-3.18) | 0.881 |
|  |  |  |  |  | 0.004 | 0.78 (0.21-2.93) | 0.717 |
|  |  |  |  |  | 0.004 | 0.63 (0.18-2.24) | 0.475 |
| [rs189437718](https://www.ebi.ac.uk/gwas/variants/rs189437718) | [Metazoa_SRP](https://www.ebi.ac.uk/gwas/genes/Metazoa_SRP), [ST3GAL1-DT](https://www.ebi.ac.uk/gwas/genes/ST3GAL1-DT) | 0.01 | 20 | 3.60x10^-7^ | 0.002 | 1.13 (0.23-5.58) | 0.881 |
|  |  |  |  |  | 0.002 | 0.81 (0.17-3.96) | 0.796 |
|  |  |  |  |  | 0.002 | 0.83 (0.18-3.95) | 0.820 |
| [rs72654815](https://www.ebi.ac.uk/gwas/variants/rs72654815) | [EIF4G3](https://www.ebi.ac.uk/gwas/genes/EIF4G3) | 0.03 | 9 | 6.79 x 10^-7^ | 0.026 | 0.67 (0.40-1.11) | 0.116 |
|  |  |  |  |  | 0.026 | 0.66 (0.38-1.12) | 0.122 |
|  |  |  |  |  | 0.026 | 0.68 (0.39-1.18) | 0.169 |
| [rs77876049](https://www.ebi.ac.uk/gwas/variants/rs77876049) | [CLMP](https://www.ebi.ac.uk/gwas/genes/CLMP), [HSPA8](https://www.ebi.ac.uk/gwas/genes/HSPA8) | 0.05 | 4 | 9.04 x 10^-6^ | 0.047 | 1.21 (0.84-1.75) | 0.296 |
|  |  |  |  |  | 0.047 | 1.21 (0.82-1.79) | 0.330 |
|  |  |  |  |  | 0.047 | 1.19 (0.81-1.75) | 0.383 |

* SNP not found in MGI dataset

** Study by Henao-Martinez *et al*., 2013 used Serum Creatinine (mg/dL) as a marker of renal function (ie: continuous variable), therefore, direct comparison of effect size to our logistic regression, which used KDIGO-staged AKI (ie: binary, categorical variable) is not possible.

Studies replicated at *P* < 0.05 threshold are denoted in **bold**.

Abbreviations: CHF = congestive heart failure; Chr = Chromosome; COPD = chronic obstructive pulmonary disease; GWAS = genome-wide association study; KDIGO = Kidney Disease: Improving Global Outcomes; MAF = Minor Allele; MGI = Michigan Genomics Initiative OR = Odds Ratio; Pos = Position; Frequency; S-AKI = Sepsis-associated Acute Kidney Injury; SOFA = Sequential Organ Failure Assessment; SE = Standard Error; SNP = single nucleotide polymorphism.
