# Supplementary material for: Genetic variants associated with sepsis-associated acute kidney injury
Source: PLoS One. 2024 Dec 5;19(12):e0311318. doi: 10.1371/journal.pone.0311318 (PMC11620412; doi:10.1371/journal.pone.0311318)
Supplement: S2 Table — A. Model 1: GWAS Performed on Primary Outcome (KDIGO Stage 2 or 3)—Controlling for age, sex, chip, and first 5 principal components. B. Model 2: GWAS Performed on Primary Outcome (KDIGO Stage 2 or 3)—Controlling for age, sex, chip, baseline serum creatinine, and first 5 principal components. C. Model 3: GWAS Performed on Primary Outcome (KDIGO Stage 2 or 3)—Controlling for age, sex, chip, baseline serum creatinine, lactate, total increase in SOFA score, comorbidities: diabetes complicated/uncomplicated, CHF, liver disease, peripheral vascular disease, COPD, and cardiac arrhythmias, and first 5 principal components. Abbreviations: CHF = congestive heart failure; Chr = Chromosome; COPD = chronic obstructive pulmonary disease; GWAS = genome-wide association study; KDIGO = Kidney Disease: Improving Global Outcomes; MAF = Minor Allele; OR = Odds Ratio; Pos = Position; Frequency; SOFA = Sequential Organ Failure Assessment; SE = Standard Error; SNP = single nucleotide polymorphism. (DOCX) [file pone.0311318.s004.docx]

**S4 Table. Genome Wide Association Study (GWAS) Results: *P* < 1 x 10^-6^, minor allele frequency > 1%**

1. Model 1: GWAS Performed on Primary Outcome (KDIGO Stage 2 or 3) -- Controlling for age, sex, chip, and first 5 principal components

| **Chr** | **Pos** | **Test Allele** | **MAF** | **SNP** | **OR** | **SE** | ***P*-value** |
| --- | --- | --- | --- | --- | --- | --- | --- |
| 21 | 34,052,644 | A | 0.00348 | rs151226077 | 2,534.6567 | 1.449 | 6.28 x 10^-8^ |
| 1 | 39,353,601 | T | 0.00172 | rs187498597 | 87,218.1663 | 2.140 | 1.06 x 10^-7^ |
| 3 | 57,227,733 | C | 0.00583 | rs113307246 | 199.7274 | 1.037 | 3.22 x 10^-7^ |
| 6 | 150,126,655 | T | 0.00139 | rs543428039 | 115,411.3728 | 2.282 | 3.26 x 10^-7^ |
| 3 | 57,301,162 | CT | 0.00579 | rs554253734 | 194.5303 | 1.037 | 3.76 x 10^-7^ |
| 7 | 15,992,306 | T | 0.26757 | rs34006964 | 1.9033 | 0.129 | 5.54 x 10^-7^ |
| 1 | 232,341,507 | C | 0.01895 | rs148779318 | 10.9088 | 0.478 | 5.83 x 10^-7^ |
| 17 | 69,432,937 | C | 0.01292 | rs117567213 | 22.8233 | 0.626 | 5.89 x 10^-7^ |
| 7 | 15,980,850 | C | 0.28144 | rs13236237 | 1.8631 | 0.125 | 6.88 x 10^-7^ |
| 6 | 70,441,352 | T | 0.00771 | rs150666240 | 79.5257 | 0.884 | 7.38 x 10^-7^ |
| 3 | 57,615,491 | C | 0.00566 | rs531586225 | 163.3358 | 1.029 | 7.39 x 10^-7^ |
| 21 | 34,072,365 | A | 0.00440 | rs79240694 | 433.6733 | 1.228 | 7.57 x 10^-7^ |
| 7 | 15,991,208 | T | 0.27122 | rs62440774 | 1.8782 | 0.128 | 7.68 x 10^-7^ |
| 2 | 229,244,823 | A | 0.00103 | rs777298734 | 1,733,472.7760 | 2.906 | 7.69 x 10^-7^ |
| 3 | 57,666,968 | C | 0.00377 | rs185743946 | 641.3533 | 1.311 | 8.15 x 10^-7^ |
| 7 | 15,991,004 | T | 0.27152 | rs144567192 | 1.8762 | 0.128 | 8.17 x 10^-7^ |
| 7 | 15,997,528 | T | 0.27162 | rs35967745 | 1.8732 | 0.128 | 8.68 x 10^-7^ |
| 1 | 39,642,710 | G | 0.00120 | rs748758604 | 711,069.3919 | 2.744 | 9.08 x 10^-7^ |
| 3 | 57,769,482 | T | 0.00551 | rs112568690 | 161.0255 | 1.036 | 9.38 x 10^-7^ |
| 4 | 188,458,366 | C | 0.30413 | rs372298 | .5515 | 0.121 | 9.56 x 10^-7^ |
| 7 | 15,997,450 | G | 0.27197 | rs17169201 | 1.8647 | 0.127 | 9.82 x 10^-7^ |

1. Model 2: GWAS Performed on Primary Outcome (KDIGO Stage 2 or 3) -- Controlling for age, sex, chip, baseline serum creatinine, and first 5 principal components

| **Chr** | **Pos** | **Test Allele** | **MAF** | **SNP** | **OR** | **SE** | ***P*-value** |
| --- | --- | --- | --- | --- | --- | --- | --- |
| 9 | 84,369,833 | A | 0.00317 | rs11140550 | 26,061.1486 | 1.992 | 3.30 x 10^-7^ |
| 9 | 84,369,848 | T | 0.00317 | rs11140551 | 26,050.0651 | 1.992 | 3.30 x 10^-7^ |
| 10 | 48,929,632 | T | 0.97964 | rs2671704 | .0271 | 0.735 | 9.07 x 10^-7^ |
| 10 | 48,929,132 | A | 0.97967 | rs2377695 | .0271 | 0.735 | 9.12 x 10^-7^ |
| 10 | 48,927,949 | G | 0.97967 | rs2671703 | .0271 | 0.735 | 9.13 x 10^-7^ |
| 10 | 48,928,027 | C | 0.97967 | rs2663023 | .0271 | 0.735 | 9.13 x 10^-7^ |
| 10 | 48,928,128 | A | 0.97967 | rs2663025 | .0271 | 0.735 | 9.13 x 10^-7^ |
| 10 | 48,929,468 | G | 0.97965 | rs1877803 | .0271 | 0.735 | 9.14 x 10^-7^ |
| 10 | 48,930,286 | C | 0.97965 | rs2671706 | .0273 | 0.735 | 9.62 x 10^-7^ |

1. Model 3: GWAS Performed on Primary Outcome (KDIGO Stage 2 or 3) -- Controlling for age, sex, chip, baseline serum creatinine, lactate, total increase in SOFA score, comorbidities: diabetes complicated/uncomplicated, CHF, liver disease, peripheral vascular disease, COPD, and cardiac arrhythmias, and first 5 principal components

| **Chr** | **Pos** | **Test Allele** | **MAF** | **SNP** | **OR** | **SE** | ***P*-value** |
| --- | --- | --- | --- | --- | --- | --- | --- |
| 9 | 84,369,833 | A | 0.00317 | rs11140550 | 26,692.5197 | 1.988 | 2.93 x 10^-7^ |
| 9 | 84,369,848 | T | 0.00317 | rs11140551 | 26,679.6339 | 1.987 | 2.93 x 10^-7^ |
| 10 | 48,929,632 | T | 0.97964 | rs2671704 | 0.0227 | 0.756 | 5.52 x 10^-7^ |
| 10 | 48,929,132 | A | 0.97967 | rs2377695 | 0.0227 | 0.756 | 5.54 x 10^-7^ |
| 10 | 48,927,949 | G | 0.97967 | rs2671703 | 0.0227 | 0.756 | 5.54 x 10^-7^ |
| 10 | 48,928,027 | C | 0.97967 | rs2663023 | 0.0227 | 0.756 | 5.54 x 10^-7^ |
| 10 | 48,928,128 | A | 0.97967 | rs2663025 | 0.0227 | 0.756 | 5.54 x 10^-7^ |
| 10 | 48,929,468 | G | 0.97965 | rs1877803 | 0.0228 | 0.756 | 5.56 x 10^-7^ |
| 10 | 48,930,286 | C | 0.97965 | rs2671706 | 0.0229 | 0.756 | 5.86 x 10^-7^ |
| 10 | 48,927,190 | T | 0.01402 | rs7920772 | 47.1415 | 0.775 | 6.65 x 10^-7^ |
| 10 | 48,925,591 | A | 0.01414 | rs79333232 | 46.4358 | 0.773 | 6.92 x 10^-7^ |
| 10 | 48,927,913 | C | 0.01393 | rs75604782 | 46.7658 | 0.775 | 7.09 x 10^-7^ |
| 10 | 48,928,621 | A | 0.01394 | rs114490633 | 46.7518 | 0.775 | 7.10 x 10^-7^ |
| 10 | 48,916,744 | A | 0.01752 | rs12248022 | 28.3211 | 0.675 | 7.22 x 10^-7^ |
| 10 | 48,916,829 | G | 0.01752 | rs59439592 | 28.3211 | 0.675 | 7.22 x 10^-7^ |
| 10 | 48,916,826 | G | 0.01753 | rs199765342 | 28.3198 | 0.675 | 7.22 x 10^-7^ |
| 10 | 48,931,582 | T | 0.01393 | rs12246580 | 46.4420 | 0.775 | 7.42 x 10^-7^ |
| 10 | 48,932,063 | G | 0.01390 | rs12255560 | 46.8749 | 0.779 | 7.79 x 10^-7^ |
| 10 | 48,923,201 | A | 0.01427 | rs74133041 | 44.7354 | 0.770 | 8.03 x 10^-7^ |
| 10 | 48,922,199 | C | 0.01428 | rs74133038 | 44.7217 | 0.770 | 8.04 x 10^-7^ |
| 10 | 48,922,837 | T | 0.01428 | rs74133040 | 44.7096 | 0.770 | 8.05 x 10^-7^ |
| 10 | 48,922,544 | A | 0.01429 | rs4261205 | 44.7059 | 0.770 | 8.05 x 10^-7^ |
| 10 | 48,929,456 | T | 0.01403 | rs115578767 | 43.9584 | 0.768 | 8.42 x 10^-7^ |

Abbreviations: CHF = congestive heart failure; Chr = Chromosome; COPD = chronic obstructive pulmonary disease; GWAS = genome-wide association study; KDIGO = Kidney Disease: Improving Global Outcomes; MAF = Minor Allele; OR = Odds Ratio; Pos = Position; Frequency; SOFA = Sequential Organ Failure Assessment; SE = Standard Error; SNP = single nucleotide polymorphism.
