# Supplementary material for: Genetic variants associated with sepsis-associated acute kidney injury
Source: PLoS One. 2024 Dec 5;19(12):e0311318. doi: 10.1371/journal.pone.0311318 (PMC11620412; doi:10.1371/journal.pone.0311318)
Supplement: S3 Table — Abbreviations: logFC: The log fold change, or the change in gene expression for each unit increase; AveExpr: The average expression across all samples; t: The logFC divided by its standard error; P.Value: The raw p-value based on t from the test that logFC differs from 0. (DOCX) [file pone.0311318.s005.docx]

**S5 Table. Differential Gene Expression of Suggested Genes in Published S-AKI Models**

|  | **PMID: 32952625** | **PMID: 33863396** | | |
| --- | --- | --- | --- | --- |
| ***Gene*** | ***S-AKI*** | ***Sepsis (0.5H)*** | ***Sepsis (24H)*** | ***Sepsis (48H)*** |
| *ARL4C* | *Downregulated* | logFC: -1.50391  AveExpr: 6.809809  t: -12.445  P: 1.30 x 10^-17^ | logFC: -1.5919  AveExpr: 6.763321  t: -15.9687  P: 2.54 x 10^-22^ | logFC: -1.42328  AveExpr: 6.878734  t: -13.4232  P: 1.12 x 10^-18^ |
| *TRIB2* |  | logFC: -1.89175  AveExpr: 5.976438  t: -14.218  P: 4.78 x 10^-20^ | logFC: -1.99516  AveExpr: 5.921807  t: -16.2588  P: 1.12 x 10^-22^ | logFC: -1.80300  AveExpr: 6.056677  t: -12.4268  P: 2.52 x 10^-17^ |
| *SLAMF6* |  | logFC: -1.20914  AveExpr: 6.745668  t: -10.1746  P: 3.07 x 10^-14^ | logFC: -1.99516  AveExpr: 5.921807  t: -16.2588  P: 1.12 x 10^-22^ | logFC: -1.12994  AveExpr: 6.808412  t: -11.0349  P: 2.43 x 10^-15^ |

Abbreviations: logFC: The log fold change, or the change in gene expression for each unit increase; AveExpr: The average expression across all samples; t: The logFC divided by its standard error; P.Value: The raw p-value based on t from the test that logFC differs from 0.
